# Nova diet quality scores and risk of weight gain in the NutriNet-Brasil cohort study

**DOI:** 10.1017/S1368980023001532

**Published:** 2023-11

**Authors:** Francine Silva dos Santos, Eurídice Martinez Steele, Caroline dos Santos Costa, Kamila Tiemman Gabe, Maria Alvim Leite, Rafael Moreira Claro, Mathilde Touvier, Bernard Srour, Maria Laura da Costa Louzada, Renata Bertazzi Levy, Carlos Augusto Monteiro

**Affiliations:** 1 Department of Nutrition, School of Public Health, University of São Paulo, São Paulo 01246-904, Brazil; 2 Center for Epidemiological Research in Nutrition and Health, Department of Nutrition, School of Public Health, University of São Paulo, São Paulo, Brazil; 3 Postgraduate Program in Nutrition in Public Health, School of Public Health, University of São Paulo, São Paulo, Brazil; 4 Department of Nutrition, Federal University of Minas Gerais, Belo Horizonte, Minas Gerais, Brazil; 5 Sorbonne Paris Nord University, INSERM U1153, INRAE U1125, CNAM, Nutritional Epidemiology Research Team (EREN), Villetaneuse, France; 6 Department of Preventive Medicine, School of Medicine, University of São Paulo, São Paulo, Brazil

**Keywords:** Food processing, Diet quality metrics, BMI, Cohort studies, Brazil

## Abstract

**Objective:**

To assess the prospective association of two diet quality scores based on the Nova food classification with BMI gain.

**Design:**

The NutriNet-Brasil cohort is an ongoing web-based prospective study with continuous recruitment of participants aged ≥ 18 years since January 2020. A short 24-h dietary recall screener including ‘yes/no’ questions about the consumption of whole plant foods (WPF) and ultra-processed foods (UPF) was completed by participants at baseline. The Nova-WPF and the Nova-UPF scores were computed by adding up positive responses regarding the consumption of thirty-three varieties of WPF and twenty-three varieties of UPF, respectively. Participants reported their height at baseline and their weight at both baseline and after approximately 15 months of follow-up. A 15-month BMI (kg/m^2^) increase of ≥5 % was coded as BMI gain.

**Setting:**

Brazil.

**Participants:**

9551 participants from the NutriNet-Brasil cohort.

**Results:**

Increasing quintiles of the Nova-UPF score were linearly associated with higher risk of BMI gain (relative risk Q5/Q1 = 1·34; 95 % CI 1·15, 1·56), whereas increasing quintiles of the Nova-WPF score were linearly associated with lower risk (relative risk Q5/Q1 = 0·80; 95 % CI 0·69, 0·94). We identified a moderate inverse correlation between the two scores (–0·33) and a partial mediating effect of the alternative score: 15 % for the total effect of the Nova-UPF score and 25 % for the total effect of the Nova-WPF score.

**Conclusions:**

The Nova-UPF and Nova-WPF scores are independently associated with mid-term BMI gain further justifying their use in diet quality monitoring systems.

The Nova system classifies all foods and food products according to the extent and purpose of the industrial processing to which they were subjected into four groups: unprocessed or minimally processed foods, processed culinary ingredients, processed foods, and ultra-processed foods (UPF)^(1,2)^.

The metric most frequently used in studies that have employed Nova to assess the quality of diets and their impact on health is the dietary share, in calories or grams, of UPF^3–5^. These are formulations of processed food substances (e.g. oils, fats, sugars, starch and protein isolates) and cosmetic additives (e.g. flavourings, colourings and emulsifiers) created by the industry as profitable and attractive alternatives to unprocessed or minimally processed foods and their culinary preparations^(2)^. Various UPF attributes of acting through known, plausible, or suggested physiologic and behavioural mechanisms relate them to several chronic diseases including the risk of overweight and obesity^(3,4)^. Furthermore, a 4-week crossover randomised clinical trial, with twenty weight-stable adults, showed that, compared to a diet with no UPF, a diet with 83 % of energy from UPF caused a substantial increase in *ad libitum* calorie intake and consequent weight gain^(5)^.

Although less often, the dietary share of Nova unprocessed/minimally processed foods has also been used to assess the quality of diets and their impact on health. These foods include unprocessed plant- and animal-sourced foods and those that were submitted to removal of inedible or undesired parts, chilling, freezing, pasteurisation, fermentation, boiling, drying, grinding and other processes that do not add salt, sugar, fat, or any other food substance to the original foods^(1)^. One cohort study observed that the dietary contribution of Nova unprocessed/minimally processed foods was inversely associated with overweight risk^(6)^. Furthermore, it is well established that a diet based on a variety of unprocessed or minimally processed foods, predominantly plants, has a protective effect on health including the prevention of overweight/obesity and other obesity-related diseases^(7)^.

However, the estimation of the dietary contribution of Nova groups requires detailed knowledge of total food intake which demands complex, high-cost and time-consuming data collection instruments such as 24-h dietary recalls. Aiming for simpler metrics that could be used in surveillance systems, authors of the Nova classification designed a short 24-h dietary recall screener that enables the calculation of two scores to capture the consumption of unprocessed or minimally processed whole plant foods (WPF) and UPF. The current study aims to assess the association of these two Nova diet quality scores with weight gain in the NutriNet-Brasil cohort study.

## Methods

### Study participants

Participants are enrolled at NutriNet-Brasil, an ongoing web-based cohort study with continuous recruitment of residents aged ≥18 years from all Brazilian regions since 26 January 2020. The NutriNet-Brasil primary aim is to investigate the prospective association between characteristics of the diet and health outcomes related to chronic non-communicable diseases in Brazil^(8)^. The study, approved by the ethics committee of the School of Public Health from São Paulo University (process No. 88455417.8.0000.5421), recruits volunteers mainly through campaigns using traditional media (e.g. television, radio, newspapers and institutional websites), as well as social medias of digital influencers and the ones created for the study. More than 100 000 volunteers have already registered at the study digital platform (https://nutrinetbrasil.fsp.usp.br/). In a web interface, volunteers answer baseline and follow-up questionnaires. These include web-based 24-h dietary recalls, alternating the short screener and the complete recall, as well as questionnaires about health status (including weight and height) and aspects potentially associated with diet and health, such as sociodemographic characteristics, physical activity, smoking, and other health-related behaviours.

### Dietary assessment

The short Nova 24-h dietary recall screener (hereinafter Nova 24-h screener) used in the current study is a low-burden survey questionnaire including checkbox format questions about the consumption of selected food items on the previous day^(9)^. These food items include thirty-three varieties of unprocessed or minimally processed WPF (vegetables excluding potatoes and cassava, fruits excluding fruit juice, wholegrain cereals, pulses, and unsalted nuts and seeds) and twenty-three varieties of UPF (drink products such as soft drinks and flavoured dairy drinks, snack products such as packaged chips and sweetened cereal bars, and ready meals such as instant noodles and reconstituted meat products, and food products that commonly accompany main meals such as salad dressings) (Table 1). Varieties of WPF and UPF were those with the greatest energy contributions to the Brazilian diet as informed by a national population-based dietary survey^(10)^.

**Table 1:** Food items considered in NOVA diet quality scores

Nova-WPF score (33 food items)	Nova-UPF score (23 food items)
Whole vegetables: lettuce; chard; watercress; arugula; collard greens; cabbage; broccoli; spinach; any other dark green; tomato; cucumber; carrot; beetroot; pumpkin; zucchini; eggplant; okra; and any other whole vegetable (18 items)	Ultra-processed snacks: packaged chips or crackers; cookies; cake snacks; cereal bars; ice cream or popsicle; chocolate bar or candies; and sugared breakfast cereals (7 items).
Whole fruits: banana; orange or tangerine; mango; papaya; pineapple; watermelon or melon; apple or pear; grape; acai; any other whole fruit (10 items)	Ultra-processed drinks: regular or diet soda; canned or bottled fruit juice (Del Valle^*®*^-type); powdered drink mix (Tang^®^-type); chocolate-flavoured drink (Nescau^®^-type); tea-based beverage (ice tea-type); and fruit- or chocolate-flavoured yogurt (6 items).
Wholegrain cereals: brown rice, maize on the cob and steel-cut oats (3 items) Whole pulses: beans, lentils or peas (1 item) Whole nuts: any type of whole nuts (1 item)	Other ultra-processed products: sausage, hamburger or nuggets; ham, salami or mortadella; loaf, hot dog, or hamburger bun; margarine; French fries (either frozen or from restaurant chains such as McDonald’s^®^); mayonnaise, ketchup or mustard; store-bought salad dressing; instant noodles (Miojo^®^-type) or packaged soup; pizza (either frozen or from restaurant chains, such as Pizza Hut^®^ or Domino’s^®^); and frozen lasagna or other frozen ready-made meals (10 items).

The current study used the Nova 24-h screener completed by participants at baseline, on three non-consecutive days over the period of 2 weeks (2 weekdays and 1 weekend day), starting on the same day they register at the study platform. Based on the responses to each screener, two diet quality scores were calculated: the Nova score of WPF (Nova-WPF score) and the Nova score of UPF (Nova-UPF score).

The Nova-WPF score was computed by adding up positive responses regarding the day before consumption of each of the thirty-three varieties of unprocessed or minimally processed WPF (therefore ranging from zero to thirty-three), whereas the Nova-UPF score was computed by adding up positive responses regarding the day before consumption of the twenty-three varieties of UPF (therefore ranging from zero to twenty-three). The complete list of the food items included in each score is described in Table 1. For this study, we averaged the scores from each of the three completed screeners, to obtain the baseline Nova-WPF and Nova-UPF participant scores.

The Nova-UPF score had been previously validated in a clientele of primary health services from the city of São Paulo, with a similar agreement^(11)^. A validation study of both scores was conducted in a random sample of 812 NutriNet-Brasil cohort participants using a full 24-h recall as the reference dietary intake method. The prevalence-adjusted and bias-adjusted kappa (Pabak) index^(12)^ was used to assess the degree of agreement in the classification of participants according to the quintiles of the dietary energy share of WPF and UPF in the diet (provided from the 24-h recall) and quintiles of the Nova diet quality scores. The Pakab index with values greater than 0·80 indicates an almost perfect agreement: 0·61 to 0·80, a substantial agreement; 0·41 to 0·60, moderate; 0·21 to 0·40, fair; and equal to or less than 0·20, slight agreement^(13)^. The validation study available in preprint version^(14)^ showed substantial agreement between participants classified, alternatively, across quintiles of the Nova-WPF scores and quintiles of the dietary energy share of all WPF (Pabak index of 0·72, 95 % CI 0·64, 0·81) as well as between participants classified across quintiles of the Nova-UPF score and quintiles of the energy share of all UPF (Pabak index of 0·79, 95 % CI 0·69, 0·88)^(14)^.

### BMI change assessment

Participants self-reported their height in metres with two decimal points at baseline. At both the baseline and approximately 15 months after baseline (mean 14·9 (±1·2) months; min. 11·0 to max. 18·0 months), participants also self-reported the date they measured their weight for the last time and their weight measured in kg. The BMI in each date was calculated as the weight in kilograms divided by the height in squared metres (kg/m²)^(15)^. The relative BMI change in the period between the two dates, expressed as a percentage of the initial BMI, was then standardised for the exact period of 15 months.

### Covariate assessment

Data on demographics, socio-economic characteristics and health behaviours were self-reported by the participants at the baseline. The baseline covariates included sex (male/female), age (years, continuous), schooling level (0–11/≥ 12 years of schooling), region of residence in Brazil (North/Northeast/Midwest/Southeast/South), smoking status (never/former/current), diet for weight loss (no/yes) and physical activity (insufficiently active/active). Physical activity was assessed with the Global Physical Activity Questionnaire (GPAQ). According to GPAQ’s data processing guidelines, we summed the time spent on work, transport, and leisure-time physical activity and classified using the cut-off points of 150 min of moderate-intensity physical activity, 75 min of vigorous-intensity physical activity, or combination of moderate- and vigorous-intensity physical activity achieving at least 600 MET-minutes/week. We consider insufficiently active participants below these cut-off points and active those with physical activity values equal to or greater than those of the cut-off points^(16)^.

### Data analysis

Data were extracted for analysis on January 2022 and included 12 074 participants who had completed the Nova 24-h screener at baseline in three non-consecutive days and had self-reported their height at baseline and their weight and date they measured their weight for the last time both at baseline and at the 15th month of follow-up. We excluded participants who reported weight measured more than 2 months before baseline or more than 6 months before the 15-month follow-up questionnaire (*n* 1690), women who were pregnant at baseline or became pregnant during the follow-up period (*n* 355), participants with implausible initial or 15-month BMI values (<15 or ≥60 kg/m²) (*n* 20), outliers for 15-month BMI change (< 0·1 centile (–10·19 kg) or > 99·9 centile (+7·63 kg), (*n* 15)) and participants with incomplete data for covariates (*n* 443).

We first described the distribution of the covariates across quintiles of each Nova dietary quality score and tested the differences using Pearson’s χ^2^ test.

The association between each Nova dietary quality score and the 15-month change in BMI (kg/m^2^) was first assessed using restricted cubic spline linear regression with knots at the 10th, 50th and 90th centiles of the Nova score distribution^(17)^ with adjustment for all the previously mentioned study covariates. The statistical significance of linear and non-linear terms was evaluated using Wald tests.

Then, Poisson crude and adjusted regression models with robust variance were used to assess the association between quintiles of each Nova diet quality score and the risk of 15-month BMI gain of 5 % or more of the initial BMI.

Two adjusted models were used to test the associations between the quintiles of each score and BMI gain: one including all the study covariates and an additional model also adjusting for the quintiles of the alternative score. Mediation analysis conducted with the *medsem* command in Stata was used to investigate the proportion of the total effect of each Nova diet quality score on the risk of 15-month BMI gain that could be explained by the alternative Nova score^(18)^.

All statistical analyses were conducted using Stata (Stata Corp, College Station Texas) version 14.0; a *P*-value of <0·05 was considered statistically significant.

## Results

Table 2 presents the baseline characteristics of participants included in the analysis (*n* 9551), overall and according to quintiles of the Nova dietary quality scores. Higher Nova-WPF scores were significantly associated with older age, higher education (≥12 years of schooling), not currently smoking, not being on weight loss diet, physically active status and having lower BMI at the baseline. Higher Nova-UPF scores were significantly associated with being male and younger, having lower education (<12 years of schooling), being insufficiently active, having higher BMI at the baseline, and living in the more economically developed South and Southeast Brazilian regions. An inverse correlation was observed between the Nova-WPF score and the Nova-UPF score at the baseline (Spearman’s coefficient = –0·33; *P*-value < 0·0001) (data not shown).

**Table 2:** Distribution (%) according to baseline sociodemographic and behavioural characteristics, and BMI intervals, overall and across quintiles of Nova dietary quality scores. Participants of the NutriNet-Brasil cohort study (2020–2022) (*n* 9551)

		Quintiles* (Q) of the Nova-WPF score						Quintiles† (Q) of the Nova-UPF score					
	Overall	Q1	Q2	Q3	Q4	Q5		Q1	Q2	Q3	Q4	Q5	
*n*	9551	1862	1757	2265	1676	1991		2139	1885	1738	1956	1833	
Variables	%	%	%	%	%	%	*P*-value	%	%	%	%	%	*P*-value
Sex							0·59						< 0·001
Men	22·2	19·3	18·6	23·2	16·9	22·0		20·3	16·2	17·5	22·5	23·4	
Women	77·8	19·6	18·4	23·9	17·7	20·5		23·0	20·7	18·4	19·9	18·0	
Age (years)							< 0·001						< 0·001
18–29	15·2	30·0	22·4	22·8	12·1	12·7		15·8	17·0	15·7	25·1	26·5	
30–39	27·4	23·1	19·2	24·9	17·3	15·5		18·7	19·3	20·0	20·4	21·6	
40–49	23·7	21·1	20·2	23·8	17·1	17·8		21·3	19·8	18·6	20·9	19·4	
50–59	19·3	13·3	16·1	24·8	19·2	26·6		28·2	22·1	17·3	18·2	14·2	
≥60	14·4	7·2	12·8	20·8	22·3	36·9		30·5	20·3	17·8	18·2	13·2	
Macro-region of residence							0·26						< 0·001
North	2·4	22·0	18·5	23·3	16·0	20·3		28·9	20·7	14·7	18·1	17·7	
Northeast	9·3	22·1	18·3	22·3	17·0	20·3		23·6	23·4	17·5	19·5	16·1	
Midwest	7·6	18·8	19·0	24·0	18·4	19·8		21·8	19·6	18·4	21·1	19·1	
Southeast	62·7	19·9	18·4	23·7	17·5	20·5		20·7	18·5	18·0	20·1	22·7	
South	17·9	16·6	18·0	24·4	18·0	23·0							
Educational level (years of schooling)							< 0·001						< 0·001
0–11	12·2	27·0	21·7	20·4	13·9	17·0		18·6	16·5	17·7	22·2	25·0	
≥12	87·8	18·5	17·9	24·2	18·1	21·4		22·9	20·2	18·3	20·2	18·4	
Smoking status							< 0·001						0·18
Never	76·3	19·7	19·0	24·2	17·2	20·0		22·2	19·9	18·2	20·5	19·3	
Former	19·0	16·8	15·9	22·3	19·6	25·4		24·2	19·7	17·9	20·3	17·9	
Current	4·7	27·5	18·5	22·3	14·7	17·0		18·5	18·1	20·1	21·0	22·3	
Physical activity							< 0·001						< 0·001
Insufficiently active	41·8	25·8	22·2	23·0	14·4	14·6		18·2	18·5	18·3	22·9	22·1	
Active	58·2	15·0	15·7	24·3	19·8	25·3		25·4	20·6	18·2	18·8	17·1	
Diet for weight loss							0·01						0·10
No	84·4	19·4	18·0	23·6	17·6	21·5		22·1	19·6	18·0	20·6	19·6	
Yes	15·6	20·2	20·3	24·3	17·6	17·6		23·8	20·4	19·2	19·6	17·0	
BMI (kg/m²)							< 0·001						< 0·001
<18·5	2·8	24·3	18·7	20·6	14·2	22·1		20·97	20·6	14·61	20·97	22·85	
18·5–24·9	49·3	15·7	16·6	23·9	18·7	25·1		26·46	21·07	18·4	18·23	15·83	
25–29·9	30·6	20·3	18·4	24·7	17·3	19·3		20·33	19·82	17·52	21·83	20·5	
≥30	17·3	28·2	23·5	21·8	15·2	11·3		14·67	15·64	19·39	24·42	25·88	

WPF, whole plant food; UPF, ultra-processed food.

*P*-values were estimated through Pearson’s χ^2^.

* Nova-WPF score (mean (min.–max.)): Q1 (2·4 (0·0–3·3)); Q2 (4·2 (3·7–4·7)); Q3 (5·7 (5·0–6·3)); Q4 (7·3 (6·7–8·0)); Q5 (10·3 (8·3–22·0)).

† Nova-UPF score (mean (min.–max.)): Q1 (0·4 (0·0–0·7)); Q2 (1·2 (1·0–1·3)); Q3 (1·8 (1·7–2·0)); Q4 (2·6 (2·3–3·0)); Q5 (4·3 (3·3–13·7)).

**Fig. 1:**
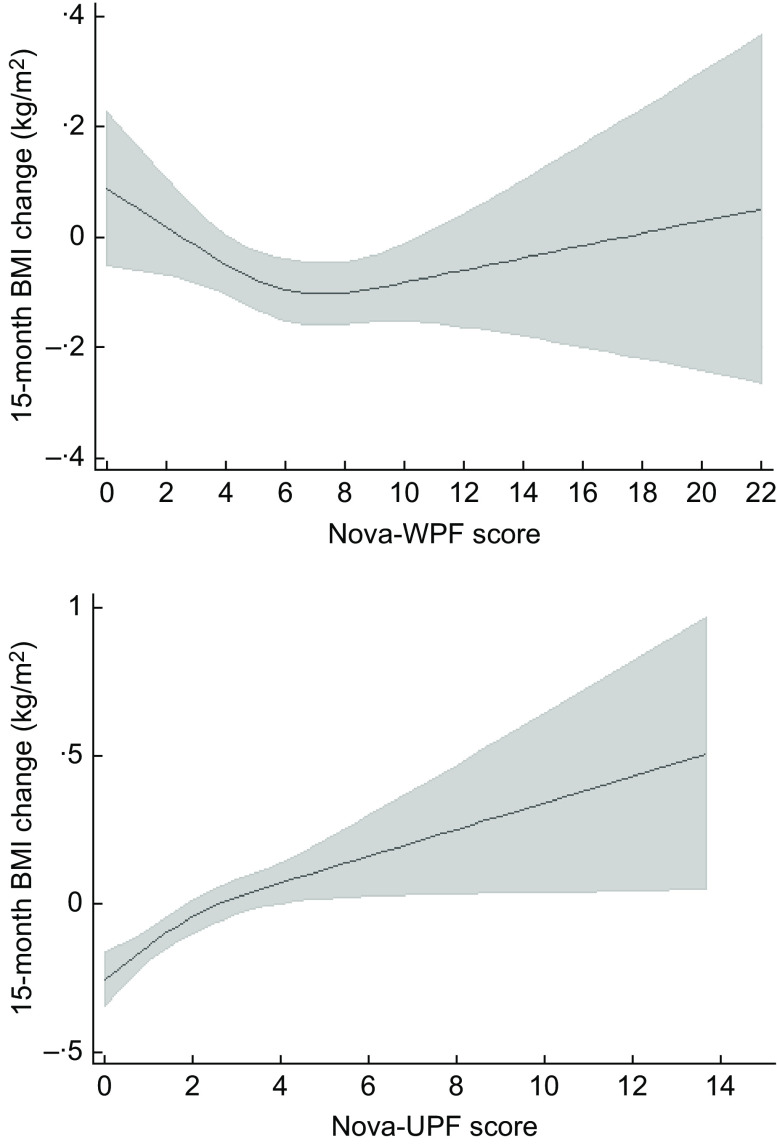
The 15-month BMI change (kg/m^2^) regressed on the baseline Nova-WPF and Nova-UPF scores using adjusted restricted cubic spline linear regression. Participants of the NutriNet-Brasil Cohort study, 2020–2022, (*n* 9551)^a^. WPF, whole plant food; UPF, ultra-processed food. ^a^Nova-WPF score: Wald test for linear term *P* < 0·01; Wald test for all non-linear terms *P* = 0·03; values corresponding to the 10th, 50th and 90th centiles knots were 2·7, 5·7, and 10·0, respectively. Nova-UPF score: Wald test for linear term *P* < 0·001; Wald test for all non-linear terms *P* = 0·12; values corresponding to the 10th, 50th and 90th centiles knots were 0·33, 1·67 and 4·0, respectively. Adjustment for sex, age (continuous), macro-region of residence, educational level, baseline smoking status, physical activity, diet for weight loss and BMI (continuous)

Restricted cubic splines linear regression models showed an inverse association between the Nova-WPF score and 15-month BMI change (kg/m^2^) (with slight departure from linearity) and a direct dose–response association in the case of the Nova-UPF score (with no departure from linearity) (Fig. 1). Supplementary Table 1 presents the results of the restricted cubic splines for all analysis models.

A 15-month BMI gain of ≥5 % of the initial BMI was observed in 16·7 % of participants (CI95 % 16·0; 17·5). Table 3 shows results from crude and adjusted regression analysis between quintiles of each Nova diet quality score and the risk of BMI gain. Increases in quintiles of the Nova-UPF score and decreases in quintiles of the Nova-WPF score were both directly and linearly associated with increased risk of BMI gain in all models including the models with adjustment for the alternative score. The protective effect of the Nova-WPF score on the risk of BMI gain was more strongly attenuated with the adjustment for the Nova-UPF score than the opposite effect of the Nova-UPF score when adjusting for Nova-WPF score. Mediation analysis showed that 15·0 % of the total effect of the Nova-UPF score on the risk of 15-month BMI gain could be explained by the Nova-WPF score, and 25·7 % of the total effect of the Nova-WPF score could be explained by the NOVA-UPF score (data not shown).

**Table 3:** Frequency (%) and relative risk (RR) of 15-month BMI gain (≥5 %) according to quintiles (Q) of baseline Nova diet quality scores. Participants of the NutriNet-Brasil cohort study (2020–2022) (*n* 9551)

NOVA diet quality scores	BMI gain (%)	Crude	95 % CI	RR		RR	
				Adjusted‡	95 % CI	Adjusted§	95 % CI
NOVA-WPF score*							
Q1	21·59	1·00		1·00		1·00	
Q2	18·16	0·84	0·74, 0·96	0·89	0·78, 1·01	0·91	0·80, 1·04
Q3	15·67	0·73	0·64, 0·83	0·79	0·69, 0·90	0·82	0·72, 0·93
Q4	15·16	0·70	0·61, 0·81	0·80	0·69, 0·92	0·84	0·73, 0·98
Q5	13·46	0·62	0·54, 0·72	0·74	0·64, 0·86	0·80	0·69, 0·94
*P*-value for linear trend		< 0·001		< 0·001		< 0·01	
NOVA-UPF score†							
Q1	13·28	1·00		1·00		1·00	
Q2	14·96	1·13	0·97, 1·31	1·08	0·93, 1·26	1·07	0·92, 1·25
Q3	17·03	1·28	1·10, 1·49	1·22	1·04, 1·41	1·18	1·02, 1·38
Q4	18·25	1·37	1·19, 1·59	1·28	1·11, 1·48	1·23	1·06, 1·43
Q5	20·68	1·56	1·35, 1·79	1·41	1·22, 1·63	1·34	1·15, 1·56
*P*-value for linear trend		< 0·001		< 0·001		< 0·001	

WPF, whole plant food; UPF, ultra-processed food.

* Nova-WPF score (min.–max.): Q1 (0·0–3·3); Q2 (3·7–4·7); Q3 (5·0–6·3); Q4 (6·7–8·0); Q5 (8·3–22·0).

† Nova-UPF score (min.–max.): Q1 (0·0–0·7); Q2 (1·0–1·3); Q3 (1·7–2·0); Q4 (2·3–3·0); Q5 (3·3–13·7).

‡ Adjusted for sex, age (continuous), macro-region of residence, educational level, smoking status, physical activity, diet for weight loss and BMI at baseline (continuous).

§ Additionally adjusted for quintiles of the other dietary score.

## Discussion

In this large prospective study with participants from all Brazilian regions, we have shown that quintiles of new diet quality scores based on the day before intake of unprocessed or minimally processed WPF and UPF, as defined by Nova food classification system, were associated, respectively, with lower and higher risk of 15-month BMI gain of ≥ 5 %. We also identified a moderate inverse correlation between the two scores (–0·33) and the partial mediating effect of the alternative score: 25 % of the protective effect of Nova-WPF score on BMI gain could be explained by its inverse correlation with the Nova-UPF score, while 15 % of the risk effect of the Nova-UPF score could be explained by its inverse correlation with the Nova-WPF score.

Direct comparisons of these findings with other studies are not possible because this is the first study that employed the two new Nova scores to study their association with health outcomes. However, in consistency with our results, previous studies have observed a direct association between the overall dietary contribution of UPF with both short- and long-term risk for weight gain and the risk of overweight/obesity^(19)^. Some of these studies also have shown that these risks remained largely unchanged after adjusting for the dietary contribution of unprocessed/minimally processed fruit and vegetables^(6,20,21)^. Likewise, an inverse association between the dietary contribution of Nova unprocessed/ minimally processed foods and risk of overweight has been previously described^(6)^.

Several pathways may explain the independent and opposite association between each of the two Nova diet quality scores and the risk of BMI gain. One possible route is the well-known positive effects of WPF on dietary parameters critical to weight gain^(21)^ and the more recently demonstrated negative effects of UPF on energy density, sugar, saturated fat, Na, fibre, and protein contents^(22,23)^, non-nutrient bioactive compound intakes^(24)^ and total water intake^(25)^. Lower or greater exposure to a modified food matrix^(26)^, cosmetic food additives^(27)^, trans fatty acids^(28)^, plastic packaging contaminants^(29)^, or neo-formed processing contaminants^(30)^, and a disturbed gut microbiota profile and integrity^(31)^ may be additional pathways explaining the opposite associations of BMI gain with the Nova-WPF and the Nova-UPF scores.

The persistent effect of each score on the risk of BMI gain after adjusting for the alternative score indicates that strategies to prevent weight gain should promote diets that are simultaneously rich in whole plant foods and reduced in UPF. These results highlight the importance of including both scores in surveillance systems that monitor population diet quality.

The strengths of this study relate to its prospective design and large sample size, as well as the baseline assessment in three non-consecutive days of the intake of WPF and UPF using a data collection tool specifically developed to capture the consumption of these foods in Brazil.

There are also some limitations that should be considered in the interpretation of our findings. This study was undertaken on volunteer participants of a cohort study on diet and health that may have a more health-conscious behaviour than the general population including more homogenous and heathier diets. This could have determined the lower contrast between extreme quintiles of each score observed in this study as compared with the overall Brazilian population^(32,33)^. This lower contrast would tend to reduce the magnitude of the association of each score with BMI gain.

The self-reporting of anthropometric data may have introduced classification bias regarding BMI gain. However, a previous similar study with French participants observed a high concordance between web-based self-reported and objectively measured weight and height^(34)^. Among the Brazilian population aged 18 years or older, self-reported measurements of weight and height were shown to be a valid method to estimate anthropometric data^(35)^.

Furthermore, non-differential social desirability bias may increase the chances of overestimating Nova-WPF or underestimating Nova-UPF scores, as well as underestimating the self-reported weight gain, biasing the studied associations towards the null.

Finally, given the observational design of the study, residual confounding arising from unadjusted factors or imprecise measurement of self-reported covariates cannot be ruled out, limiting our capacity to make causal inferences.

## Conclusions

The current study shows that Nova-UPF and Nova-WPF scores are independently associated with mid-term BMI gain further justifying the use of both scores in diet quality monitoring systems.

## Supplementary material

For supplementary material accompanying this paper visit https://doi.org/10.1017/S1368980023001532. click here to view supplementary material
