# Supplementary material for: Nova diet quality scores and risk of weight gain in the NutriNet-Brasil cohort study
Source: Public Health Nutr. 2023 Jul 31;26(11):2366–73. doi: 10.1017/S1368980023001532 (PMC10641608; doi:10.1017/S1368980023001532)
Supplement: Supplementary file 1 [file S1368980023001532sup001.docx]

**Supplementary Table 1.** The 15-month BMI change (kg/m2) regressed on the baseline Nova diet quality scores using restricted cubic spline linear regression. Participants of the NutriNet-Brasil Cohort study, 2020-2022, (n 9 551).

| NOVA diet quality scores | β (95% CI)  Crude | p-value | β (95% CI)  Adjusted^a^ | p-value | β (95% CI)  Adjusted^b^ | p-value |
| --- | --- | --- | --- | --- | --- | --- |
| NOVA-WPF score |  |  |  |  |  |  |
| Linear term | -0.05 (-0.08; -0.01) | 0.01 | -0.05 (-0.08; -0.02) | <0.01 | -0.04 (-0.07; -0.01) | 0.02 |
| Non-linear terms | 0.04 (0.01; 0.08) | 0.02 | 0.04 (0.01; 0.08) | 0.02 | 0.04 (0.00; 0.08) | 0.04 |
| NOVA-UPF score |  |  |  |  |  |  |
| Linear term | 0.10 (0.03; 0.17) | <0.01 | 0.12 (0.06; 0.19) | <0.001 | 0.12 (0.05; 0.18) | <0.01 |
| Non-linear terms | -0.05 (-0.14; 0.04) | 0.26 | -0.07 (-0.16; 0.02) | 0.12 | -0.07 (-0.16; 0.02) | 0.15 |

^a^Adjusted for sex, age (continuous), macro-region of residence, educational level, smoking status, physical activity, diet for weight loss, and BMI at baseline (continuous). ^b^Additionaly adjusted for quintiles of the other dietary score.
